# Optimizing Inpatient Nutrition Care of Adult Patients with Inflammatory Bowel Disease in the 21st Century

**DOI:** 10.3390/nu13051581

**Published:** 2021-05-09

**Authors:** Elaine Chiu, Chris Oleynick, Maitreyi Raman, Barbara Bielawska

**Affiliations:** 1 Division of Gastroenterology, University of Calgary, Calgary, AB T2N 1N4, Canada; elaine.a.chiu@gmail.com; 2 Division of Internal Medicine, University of Calgary, Calgary, AB T2N 1N4, Canada; cjoleynick@gmail.com; 3 Department of Medicine, Division of Gastroenterology, University of Ottawa, Ottawa, ON K1H 8L6, Canada; bbielawska@toh.ca

**Keywords:** inflammatory bowel disease, malnutrition, nutrition support, enteral nutrition, peripheral parenteral nutrition, central parenteral nutrition, sarcopenia

## Abstract

Malnutrition is highly prevalent in inflammatory bowel disease (IBD) patients and disproportionately affects those admitted to hospital. Malnutrition is a risk factor for many complications in IBD, including prolonged hospitalization, infection, greater need for surgery, development of venous thromboembolism, post-operative complications, and mortality. Early screening for malnutrition and prompt nutrition intervention if indicated has been shown to prevent or mitigate many of these outlined risk factors. There are many causes of malnutrition in IBD including reduced oral food intake, medications, active inflammation, and prior surgical resections. Hospitalization can further compound pre-existing malnutrition through inappropriate diet restrictions, nil per os (NPO) for endoscopy and imaging, or partial bowel obstruction, resulting in “post-hospital syndrome” after discharge and readmission. The aim of this article is to inform clinicians of the prevalence and consequences of malnutrition in IBD, as well as available screening and assessment tools for diagnosis, and to offer an organized approach to the nutritional care of hospitalized adult IBD patients.

## 1. Introduction

Inflammatory bowel disease (IBD) is a chronic debilitating inflammatory disorder of the gastrointestinal tract characterized by a relapsing and remitting course. The etiology of IBD is incompletely understood, but includes an imbalance between pro-inflammatory and anti-inflammatory signaling arising from genetic susceptibility and environmental triggers [1]. The two main subtypes of IBD are Crohn’s disease (CD) and Ulcerative colitis (UC). CD can affect the entire gastrointestinal tract from mouth to anus, with transmural inflammation affecting the full thickness of the bowel that can lead to fistulization, abscess formation, stenosis, and bowel obstruction. UC universally involves the rectum with variable involvement of the colon and does not involve the small bowel or upper digestive tract. The inflammation of UC is restricted to the mucosa, except in fulminant disease. The natural history of IBD is characterized by disease flares of variable duration and severity, with periods of remission. The prevalence of IBD is increasing globally with data from 2017 suggesting that more than three million people in the United States and Europe are living with the condition, and nearly seven million are affected worldwide [2].

IBD is often diagnosed in young adulthood or middle age, resulting in a large burden of disability-adjusted life years that is now improving as more effective treatments become available [3]. Advances in immunotherapy have improved outcomes, but many of these medications are expensive, have undesirable side effects, and are not always available. Even with effective therapy, the risk of disease progression or developing complications requiring surgery remains high, with a ten-year surgical risk of 46.6% and 15.6% in CD and UC, respectively [4]. 

Malnutrition is a frequently under-recognized complication of IBD despite its high prevalence ranging between 20 and 85% [5,6]. Although undernutrition and malnutrition are often used interchangeably, a recent guideline from the European Society for Clinical Nutrition and Metabolism (ESPEN) defines malnutrition more broadly as “a state resulting from lack of intake or uptake of nutrition that leads to altered body composition (decreased fat free mass) and body cell mass leading to diminished physical and mental function and impaired clinical outcome from disease” [7]. Malnutrition can thus include those with normal or even elevated body weight.

**Figure 1:**
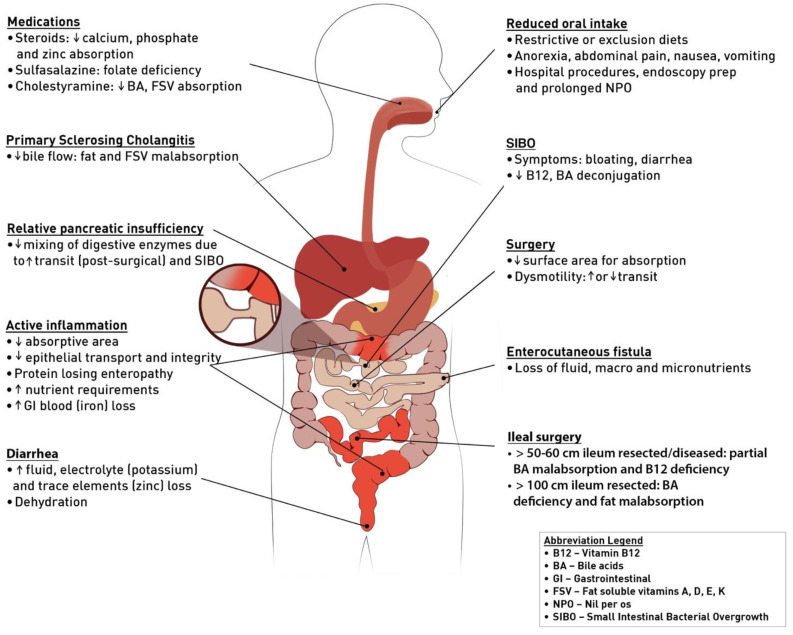
Multifactorial mechanisms of malnutrition in inflammatory bowel disease (IBD).

In the case of IBD, malnutrition can result from a number of different mechanisms (see Figure 1) including decreased oral intake, medication-related nutrient interactions, malabsorption, gastrointestinal nutrient loss, bile salt wasting, surgical resections, active inflammation, small intestinal bacterial overgrowth, chronic dehydration with resultant renal insufficiency, micronutrient deficiency, and metabolic bone disease. Malnutrition may present differently in CD compared to UC, and can often be predicted based on the anatomical location and severity of disease. Small bowel involvement in CD may lead to greater protein-energy malnutrition and micronutrient deficiencies over time, whereas UC patients tend to present with rapid nutritional decline during an acute flare or hospitalization [8,9]. Patients with fistulizing CD or those who have undergone bowel resection are at a particularly high risk of malnutrition [10,11]. 

Sarcopenia is a nutrition-associated disorder characterized by reduced muscle mass and strength. There are multiple contributors to sarcopenia in IBD, including chronic inflammation, malnutrition, and physical inactivity [12]. Sarcopenia is present in approximately 40% of patients with gastrointestinal conditions [13]. Patients with IBD who are affected by malnutrition have up to 60% reduced muscle mass compared to healthy matched controls, and the presence of malnutrition increases the risk of adverse outcomes [14]. Given the increasing prevalence of obesity among patients with IBD, elevated body mass index (BMI) frequently coexists with low muscle mass, termed sarcopenic obesity. Sarcopenia in IBD is associated with functional decline and a high risk of disability and mortality [15]. 

There is a wealth of data pertaining to malnutrition in hospitalized patients as a whole, and while some IBD-specific data exist, this area is still a growing field of interest. The prevalence of malnutrition is high among patients admitted to hospitals in general, with studies demonstrating a prevalence of up to 45% [16,17]. A large study by Tobert et al. found that only 4% of patients in American academic centers were given a diagnosis of malnutrition, suggesting significant under-recognition by clinicians [18,19]. 

There are significant adverse clinical implications of underdiagnosing malnutrition in IBD patients. We summarize the current state of knowledge and make best practice suggestions based on expert opinions that practitioners can readily apply to optimize nutritional and clinical outcomes in hospitalized IBD patients. The primary objective of this article is to serve as an expert narrative review to inform clinicians of the prevalence and consequences of malnutrition in IBD, available screening and assessment tools for diagnosis, and its use during hospitalization, and to offer an organized approach to the nutritional care of hospitalized adult IBD patients. 

We employed a rigorous search strategy for our narrative review for nutrition interventions (Section 5). We searched Ovid Medline using the search terms ((Crohn Disease) or (Colitis, Ulcerative) or (Inflammatory Bowel Disease)) and ((Nutritional Support) or (Enteral Nutrition) or (Parenteral Nutrition)). An additional search of Pubmed was also performed, and no additional articles were identified. This was limited to full-text English-language articles on humans. We discuss the most pertinent studies to guide the inpatient management of malnutrition in patients with IBD.

## 2. What Are the Clinical Implications of Malnutrition in Hospitalized IBD Patients?

Malnutrition in hospitalized patients is associated with prolonged length of stay, higher readmission rates after discharge, greater rates of complications, infections, and increased mortality [18,20,21]. Additionally, the nutritional status of patients often worsens during hospital admission for a variety of reasons including anorexia due to illness, lack of home support, and hospital protocols placing patients NPO for imaging and procedures. While these orders may be well-placed at the time, prolonged unintentional continuation of iatrogenic lack of feeding contributes to “post-hospital syndrome” [22,23]. Malnutrition places a high burden on the healthcare system, with the overall costs of hospitalized malnourished patients being up to 34% higher than those of well-nourished controls [18].

Specific to IBD, malnutrition is disproportionately higher in those admitted to hospitals [11,24]. Malnutrition in IBD has been shown to be an independent risk factor for prolonged hospital admission, increased mortality, admission after emergency room presentation, increased risk of infections, greater need for urgent or emergent surgery, and the development of venous thromboembolism during hospitalization [11,25,26,27,28]. Malnourished IBD patients receiving immunosuppressive agents may be at the greatest risk for opportunistic infections [29]. 

Patients with severe IBD frequently have restricted oral intake due to their symptoms. Long-term avoidance of specific foods was reported to be as high as 77% in a recent study and can lead to micronutrient deficiencies even in the absence of weight loss [24]. When IBD patients are admitted to a hospital, dietary restrictions happen frequently for imaging and endoscopic testing. In patients who are already malnourished, the negative implications on nutrition status are amplified, especially if hospital admissions are frequent or prolonged.

Evidence suggests that identifying malnutrition and introducing a nutritional intervention can improve outcomes in hospitalized patients as a whole [30,31]. While there are limited data regarding the impact of nutritional interventions specifically in hospitalized IBD patients, supervised interventions with nutrition-trained health professionals are likely to improve outcomes and carry minimal risk of harm. Therefore, screening and, when appropriate, interventions for malnutrition, should be undertaken in all hospitalized IBD patients.

## 3. How Can Clinicians Diagnose Malnutrition in IBD? 

Defining the malnutrition construct as it applies to IBD remains challenging, largely due to a lack of validated tools for its diagnosis. The prevalence is high, but estimates are variable due to use of different tools. An ideal gold standard would include elements of history, physical examination, anthropometric measurements, laboratory markers, body composition, and assessments of physical and mental functioning. While such a comprehensive assessment would capture the spectrum of malnutrition in IBD, it is not practical and could not be universally applied. In clinical practice and even in research settings, malnutrition in IBD is usually diagnosed using nutritional assessment tools (NAT); however, many of these are not yet comprehensively validated in IBD.

Various NAT are available and validated in general medical conditions, including bedside global assessment, bedside muscle measurements, bedside body composition analysis, and cross-sectional measures of body composition. Bedside global assessments include elements of clinical history, diet history (including food exclusions, restrictions), physical examination, and laboratory values. The Subjective Global Assessment (SGA) is one of the most widely used bedside global NAT, and has been well validated in general populations [32]. In IBD, the SGA shows significant associations with length of stay in hospitals; however, assessments missed a large proportion of patients who had decreased body cell mass as determined by bioimpedance analysis [33]. 

Bedside muscle measurements include hand grip strength (HGS), mid-upper arm circumference (MUAC), and mid-upper arm muscle circumference (MUAMC). HGS has been shown to be an effective, quick, and convenient parameter to predict functional status and muscular health in CD patients compared to healthy controls [34]. HGS correlated well with the strength of other muscle compartments with good reliability and reproducibility, and can be utilized in hospital and community settings for IBD patients [13]. MUAC is an easily obtained anthropometric measure of the right upper arm at the midpoint between the tip of the shoulder and tip of the elbow. MUAMC is more complicated and requires additional steps, as it incorporates MUAC and tricep skinfold measurements into a formula to determine the amount of muscle and bone in the upper arm. Mijac et al. identified that both MUAC and MUAMC were significantly lower in IBD patients compared to control subjects [10]. 

NAT focused on identifying altered body composition include bioelectrical impedance analysis (BIA), which can be conducted at the bedside, or radiologic tools such as dual-energy x-ray absorptiometry (DEXA), computer tomography (CT), and magnetic resonance imaging (MRI). Body composition analysis estimates lean body mass and can be used to determine the presence of sarcopenia. The practical use of DEXA and BIA are limited in a hospital environment due to extra resources including equipment and time, or potential adverse effects such as radiation. Currently, body composition analysis tools are primarily being used for research. While CT and MRI are frequently performed on patients with IBD for assessment of their disease, software for calculations of body composition from such imaging is not universally available. Thus, with resource or time constraints, HGS and MUAC are more portable and easily obtained in a hospital and outpatient environment compared to DEXA or BIA, which require specialized equipment. However, clinicians may not have the tools and time to perform these bedside measurements, which are likely most useful when taken serially.

ESPEN criteria to diagnose malnutrition require either a low BMI of <18.5 kg/m^2^ or the combined finding of unintentional weight loss with reduced BMI (<20 or <22 kg/m^2^ in subjects younger and older than 70 years, respectively) or reduced fat free mass index (FFMI) (<15 kg/m^2^ for females and <17 kg/m^2^ for males) [35]. Fat free mass (FFM) is often calculated using BIA or DEXA with FFMI obtained by dividing FFM/height^2^. The American Society of Parenteral and Enteral Nutrition (ASPEN) criteria for malnutrition require two or more characteristics including insufficient energy intake, weight loss, loss of muscle mass, loss of subcutaneous fat, and localized or generalized fluid accumulation that may sometimes mask weight loss or diminished functional status as measured by HGS [36].

However, ESPEN and ASPEN criteria may not capture all components of malnutrition in IBD, especially micronutrient deficiencies that may arise due to malabsorption, selective avoidance of food groups, or high losses. Until a gold standard for malnutrition in IBD is created, we recommend that a diagnosis of malnutrition in IBD not require a low BMI nor unintentional weight loss. We suggest if an IBD patient has a minimum of two criteria below, a diagnosis of malnutrition should be considered. These include food restrictions/avoidance, active luminal disease, symptoms of nausea/vomiting/diarrhea/poor appetite beyond one week, unintentional weight loss >5% in 3 months, HGS < 20% percentile based on age/gender, or low FFMI < 15 kg/m^2^ for females and <17 kg/m^2^ for males [35]. These broad criteria aim to identify malnutrition in IBD and capture evidence of malnutrition that may not always be reflected via weight loss or low BMI alone. 

## 4. What Tools Can We Use to Screen All IBD Patients for Malnutrition?

While a formal nutrition assessment in all inpatients would appear to be the optimal approach, this is not feasible due to limited resources. Nutritional screening tools (NST) are rapid and simple evaluation tools that can be completed by any healthcare team member, and even the patient. Screening tools are designed to detect risk for protein and energy malnutrition, and/or predict whether malnutrition is likely to occur under present or future conditions [37]. A positive NST result should prompt further investigation through validated NAT ideally administered by nutrition professionals. Major nutritional guidelines suggest that all patients admitted to the hospital should undergo screening for malnutrition [38]. ESPEN 2020 guidelines suggest that all newly diagnosed IBD patients should be screened for malnutrition [39]. In practice, this does not always occur due to under-recognition of malnutrition by healthcare professionals, lack of standardized routine screening protocols, self-perceived lack of knowledge and skills, lack of validated tools, and the lack of assignment of responsibility [40,41,42,43,44].

Familiar and validated NST include the Malnutrition Universal Screening Tool (MUST) and Nutrition Risk Screening (NRS-2002). MUST was initially developed for outpatient settings where serious confounders of the effect of malnutrition are relatively rare [37]. However, many institutions utilized MUST in hospitalized patients with good inter-rater reliability [45]. NRS-2002 was designed to detect the presence or risk of malnutrition in hospitalized settings by assessing disease severity as this may increase nutrition requirements [37].

Both the NRS-2002 and MUST factor in BMI, amount of weight loss in a specified time, any reduced nutritional intake, and assessment of whether the patient is acutely ill. Unlike MUST, the NRS-2002 incorporates an age adjustment score of ≥70 years old, outlines specific medical conditions to stratify disease severity, and quantifies ranges of reduced food intake in a one-week span. Kondrup et al. outline the specific scoring parameters of these two screening tools [37]. A recent multicenter randomized controlled study that identified hospitalized patients screened positive with NRS-2002 who received nutritional intervention found that these patients had a reduction in mortality, as well as improvements in functional status and quality of life [46]. A study by Raslan et al. showed that NRS-2002 was able to predict unfavorable clinical outcomes best in hospitalized patients [47]. Meanwhile, other studies found that MUST has good predictive validity for mortality and length of stay for hospitalized elderly patients [48].

A 2019 systematic review explored various NST used in IBD patients including MUST, NRS-2002, Malnutrition Inflammation Risk Tool (MIRT), and Saskatchewan IBD-Nutrition Risk (SaskIBD-NR tool) [49]. The systematic review supported the association of NST and NAT with relevant outcomes, but the heterogeneity called for further studies before an optimal tool could be recommended. To date, there are only two studies assessing NST in hospitalized IBD patients which both used NRS-2002 [50,51]. Takaoka et al. outlined how NRS-2002 significantly predicted hospital length of stay but did not predict need for surgery [50]. Two outpatient IBD studies were successful in screening for malnutrition using patient-administered MUST compared to healthcare provider MUST screening [52,53]. The SaskIBD-NR tool is an IBD-specific outpatient screening tool validated against a Registered Dietitian (RD)-led global nutrition assessment that does not capture disease severity. This tool was specifically designed to consider food restriction or elimination diets, and micronutrient deficiencies frequently observed in IBD patients [54]. MIRT incorporates BMI and unintentional weight loss over three months, and unlike other screening tools, incorporates the serum inflammatory marker c-reactive protein. Jansen et al. discussed how MIRT showed significant associations with CD-related days in hospital, number of flares, complications, and CD-related surgeries [55].

We recommend that all IBD patients with a new diagnosis, flare, or complication associated with their IBD, including hospitalization, infection and surgery, be screened for malnutrition. Patients with active disease have the highest risk of malnutrition, especially in newly diagnosed IBD [56]. Identifying ideal and/or validated NST for IBD patients, both for the inpatient and outpatient setting, remains a priority. Until then, using a simple yet well-validated NST with high sensitivity is most important in the acute care setting [57]. While MIRT and SaskIBD-NR show promise for future IBD nutrition screening, further validation and inter-rater reliability are required before they can be suggested for both outpatients and inpatients. Given the success of hospitalized in-patient malnutrition screening in other populations that led to nutritional interventions and reduced patient mortality and readmissions, we suggest using either the MUST or NRS-2002 to screen hospitalized IBD patients [46,48]. 

Hospitalized IBD patients screened for risk of malnutrition should have comprehensive nutritional assessments involving a dietitian and a nutritional management plan that is well-developed. Focus groups highlighted that physicians do not screen for malnutrition frequently enough and miss opportunities to intervene early in malnourished IBD patients [58]. Given the constraints in time and resources, care teams should involve a dietitian within 24 h of admission if screened for moderate to high risk of malnutrition for nutrition assessment and intervention. 

## 5. What Is the Approach to Nutrition Support in Hospitalized Patients with IBD?

**Figure 2:**
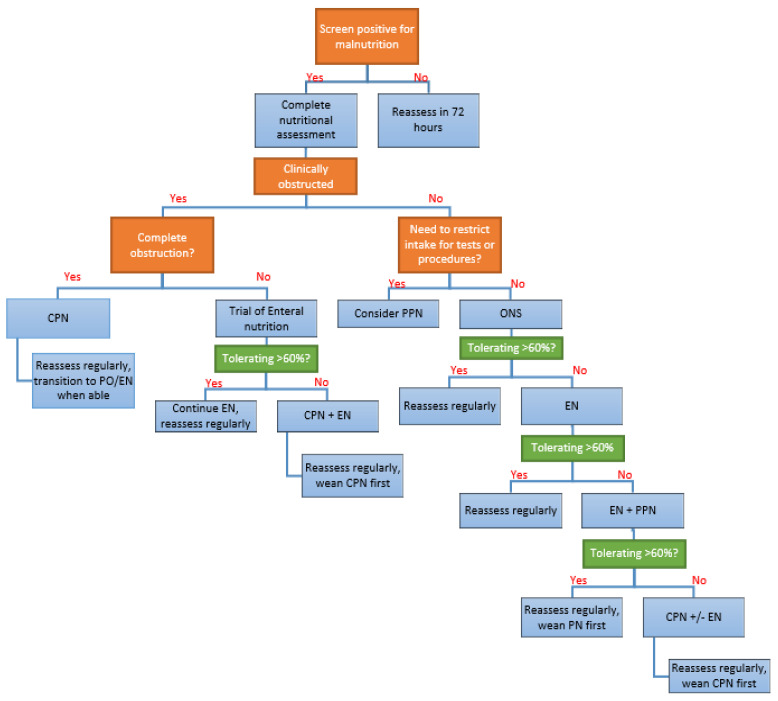
Proposed algorithm for nutrition support in hospitalized IBD patients. CPN, central parenteral nutrition; EN, enteral nutrition; ONS, oral nutritional supplement; PO, per os; PPN, peripheral parenteral nutrition.

Nutritional assessment and therapy should be initiated within 24 h of hospital admission in patients assessed as positive for malnutrition risk. Nutrition intervention can consist of one or more of: oral nutritional supplements (ONS), enteral nutrition (EN), peripheral parenteral nutrition (PPN), or central parenteral nutrition (CPN). A proposed algorithm for nutrition support is presented in Figure 2. 

### 5.1. Oral Nutrition Support

When safe and feasible, using the oral or enteral route is the preferred method for nutrition support, and oral feeding is preferred to tube feeding. Advantages of using the gut to deliver nutrition support include lower cost, avoidance of intravenous (IV) access related complications, maintenance of epithelial barrier integrity, immune modulation, and effects on the microbiome [59]. ONS are among the first nutritional interventions usually tried in hospitalized patients, but these can be inadequately tolerated due to symptoms including nausea, vomiting, and anorexia.

### 5.2. Enteral Nutrition 

EN is indicated in patients who cannot meet nutritional needs orally but have a sufficiently intact gastrointestinal tract for micro- and macronutrient absorption. Reasons for inadequate oral intake can include malabsorption, active inflammation, diarrhea, anorexia, nausea, vomiting, and abdominal pain. 

There are several formulations available for enteral (and oral) nutrition: polymeric (whole protein, complex carbohydrate, long chain triglycerides), semi-elemental (peptides, simple carbohydrates, medium chain triglycerides), and elemental (amino acids, maltodextrins and monosaccharides, fatty acids). Standard polymeric formulas are generally recommended as first-line when using EN. However, certain patients who have significant malabsorption or maldigestion can benefit from predigested semi-elemental or elemental formulas. These predigested formulas are higher in osmolarity and are not palatable, and best infused slowly using a feeding tube. 

In patients who can eat and drink but are not meeting their needs orally, supplemental EN can be used to infuse nutrients slowly at a controlled rate to reduce intolerance related to bolus feeding. Supplemental EN can also be used only overnight in order not to interfere with spontaneous daytime oral intake. Even in patients with CD who present with symptoms of partial bowel obstruction due to intestinal stricture, EN can be used if tolerated, especially if enteral access can be obtained distally to the stricture [60]. Even if the stricture cannot be bypassed, EN can still be successful in partial bowel obstructions with slowly delivered liquid nutrition at a controlled rate.

In patients with CD who present with complications requiring surgery, such as bowel resection for active or fibrostenotic disease or enterocutaneous fistula, internal fistula, or intra-abdominal abscess, there is emerging evidence regarding the benefit of pre-operative EN. In a recent case control study of patients with CD undergoing surgery, Yamamoto et al. demonstrated that compared to no nutritional intervention, a period of two or more weeks of pre-operative EN with an elemental diet was associated with a significant reduction in risk of post-operative wound and anastomotic complications [61]. In another study, patients with CD who required surgery were evaluated for the presence of malnutrition, and those with malnutrition received pre-operative EN for at least two weeks, while well-nourished patients went directly to surgery. Post-operative complications were equal in the two groups (malnourished treated with EN vs. well-nourished); however, two patients in the EN group were able to avoid surgery altogether after becoming candidates for medical therapy with biologic treatment and in the case of one patient, endoscopic dilation [59]. Systemic inflammatory markers improved in patients who received EN. In another series from China, malnourished CD patients with intra-abdominal abscess who received EN had statistically significant improvements in serum albumin and hemoglobin levels, as well as a decline in inflammatory markers, and they also were less likely to require surgery [62]. These studies all demonstrate that the nutritional status of IBD patients can be improved during EN.

While the usual indication for EN in adult hospitalized patients is to treat malnutrition, there is also evidence for its use as a primary therapy for inflammation in CD. Exclusive enteral nutrition (EEN) is an acceptable first line treatment for pediatric CD [60]. However, there is insufficient evidence in the adult population, and the efficacy is significantly limited by low palatability and compliance with this approach. Emerging evidence and a proposed approach to use EEN in adults was presented by Day et al. [63]. EEN to induce disease remission can be considered in carefully selected patients after a discussion of risks and benefits, and with clear targets in mind [63]. A detailed discussion of this topic is beyond the scope of our narrative review.

### 5.3. Peripheral Parenteral Nutrition

It is accepted that malnourished patients who cannot meet nutritional needs by the oral or enteral route should have parenteral nutrition (PN) initiated [64]. PN involves IV infusion of a sterile solution containing macronutrients, electrolytes, vitamins, and minerals. PN may be specially compounded by an institutional pharmacy, or administered through commercially available premixed solutions for central or peripheral delivery. 

PN implementation should be considered if a patient is meeting less than 50–60% of nutritional needs via the gut [60,64]. PN has traditionally been delivered by a central line, which allows for administration of a concentrated, high osmolarity solution that can completely meet nutrient, fluid, and electrolyte needs of patients. However, there has been increasing interest to utilize PPN in malnourished hospitalized IBD patients as supportive nutritional therapy. PPN involves infusion of parenteral nutrition into a peripheral vein. Unlike CPN, a central line is not required to administer PPN, which can expedite initiation of nutrition therapy in malnourished patients. Use of exclusive PPN for nutrition support may not fully meet nutritional needs due to osmolarity constraints that limit infusion capacity [65]. PPN osmolarity needs to be restricted as compared to CPN due to the occurrence of phlebitis in peripheral veins at higher osmolarities (typically above 900 mOsm/L) [66]. However, PPN can be a worthwhile nutrition intervention to explore as a short-term supportive strategy in patients with malnutrition, while an adequate nutrition plan is developed. Vigilance for common feeding complications such as refeeding syndrome risk, volume overload, and metabolic derangements are necessary even with PPN use. PPN should be used cautiously when comorbidities such as cirrhosis, heart failure, and renal impairment are present. The risk of venous injury requires regular vigilant monitoring by caregivers for signs of infiltration, phlebitis, or thrombophlebitis [65]. PPN should not be used as a sole source of nutrition support beyond 10–14 days, and if PN is required beyond 14 days to supplement oral and/or enteral feeding to ensure adequate energy and protein delivery, a central line should be placed [64]. 

Although guidelines state that there is insufficient high-quality data from well-controlled observational and randomized controlled trials to support the routine use of PPN, clinical experience points to scenarios in which PPN can be utilized to optimize nutrition for hospitalized IBD patients [64]. PPN can be advantageous in select malnourished hospitalized IBD patients enduring prolonged periods of fasting/NPO or suboptimal caloric intake in preparation for endoscopy, diagnostic imaging, or bowel obstructions. Clear fluids are devoid of significant macro- and micronutrients, contributing to iatrogenic malnutrition, and clinicians may often subject patients to NPO and clear fluid diets for prolonged time periods. Restrictive diet orders should be re-evaluated frequently to ensure they are indicated.

In such a setting of short-term partial restriction of oral intake in a malnourished patient, PPN is a logical intervention. Rapid supplementation via PPN can be expedited as a central line is not required. As delays in central line insertion can take between 24 and 72 h at some institutions, PPN can also serve as a bridge to longer-term CPN.

### 5.4. Central Parenteral Nutrition

CPN is indicated in patients with significant malabsorption, short bowel syndrome, intestinal obstruction, high output fistula, ileus, intractable vomiting, or uncontrolled severe gastrointestinal bleeding. It is also indicated in those who would benefit from PPN, as described above, but in whom PPN is contraindicated or not feasible, such as those with difficult peripheral IV access, severe malnutrition, fluid restrictions, or metabolic derangements, and those in whom the duration of PN therapy is anticipated to be more than 10–14 days. CPN can also be used in patients who meet criteria for short-term PPN use but happen to have a central line in place already. However, a lumen dedicated for PN use is recommended to reduce infection risk [64]. Even in patients with severe malabsorption or a partially obstructed bowel, enteral nutrition should always be attempted prior to PN, and used in combination with PN rather than PN alone unless there is a contraindication to enteral feeding. In a meta-analysis and meta-regression of ten trials of IBD patients who received PN in combination with food, there was demonstrated improvement in the Crohn’s Disease Activity Index (CDAI) and albumin, with a greater effect with more time [67].

## 6. How Should Nutrition Therapy Be Optimized in IBD Perioperatively? 

Malnutrition increases the risk of post-operative complications in patients with IBD, including wound and infectious complications, as well as mortality [68]. 

**Figure 3:**
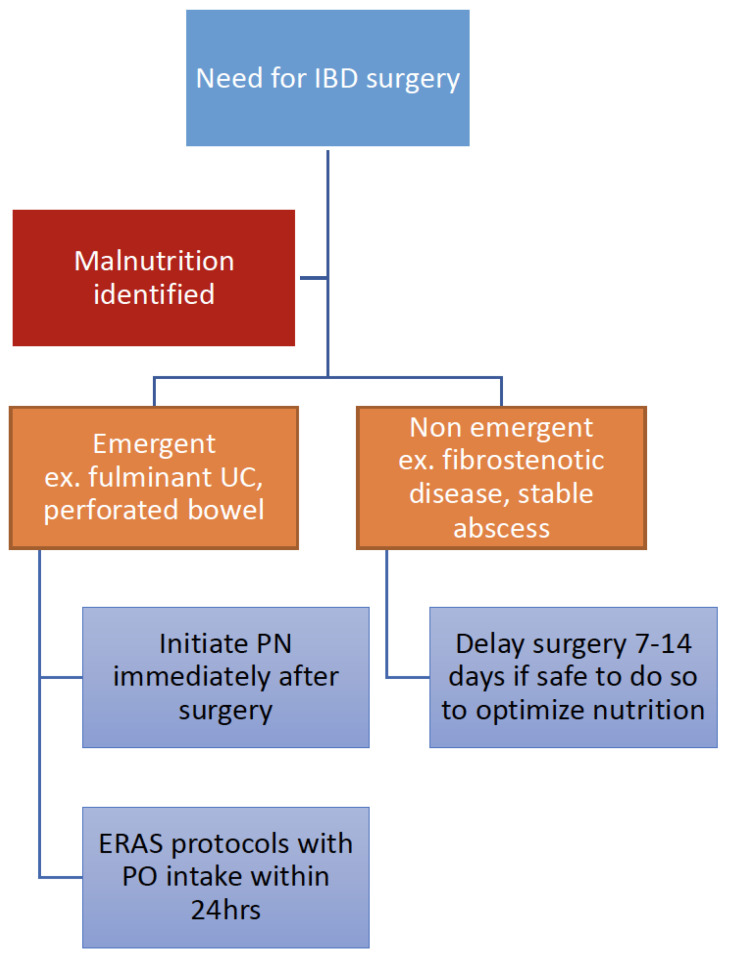
In the setting of malnutrition, IBD surgery should be delayed for 7–14 days to allow for nutritional intervention, if safe to do so, such as in the case of fibrostenotic strictures or stable intra-abdominal abscess in Crohn’s disease (CD). In the setting of emergency IBD surgery in a malnourished patient, such as with fulminant Ulcerative colitis (UC) or perforated bowel obstruction, post-operative EN and/or PN should be initiated immediately if the patient will not be able to resume full diet within 7 days of surgery. Enhanced recovery after surgery (ERAS) protocols should be applied, with oral intake and/or EN initiated within 24 h of surgery [60].

Nutritional interventions, both enteral and parenteral, were demonstrated to ameliorate this risk [59,69,70,71] As described in Section 5B, pre-operative enteral nutrition for two or more weeks was demonstrated to reduce post-operative wound and infectious complications in patients with complicated CD [59,61]. In these studies, there were reductions in inflammatory markers (CRP) and clinical severity scoring (Harvey-Bradshaw Index, HBI) [59,61]. There is a lack of high-quality data with regards to perioperative parenteral nutrition use in IBD, as retrospective studies in this field are severely limited by selection bias. However, retrospective series show reductions in CDAI, reduced post-operative complications, and reduced length of bowel resection [70]. Although there is a paucity of IBD-specific data, the impact of malnutrition on perioperative complications in the setting of other chronic inflammatory diseases is well documented and forms the basis of guidelines for perioperative nutrition support in patients with IBD [60]. Guideline-based recommendations for perioperative nutrition care are presented in Figure 3. 

Post-operative follow-up of IBD patients with intestinal resections is key to reducing the risk of new or progressive malnutrition. As outlined in Figure 1, micronutrient deficiencies can occur with ileal resection including vitamin B12, bile acid, and fat malabsorption. Bowel resections result in decreased surface area for nutrient absorption and contribute to dysmotility. Patients with long segment bowel resections and those with ileostomies can have significantly increased fluid losses and develop dehydration. Post-operative follow-up should consider the extent and location of surgery, with attention to current and expected nutritional deficiencies.

## 7. What Is the Role of Multidisciplinary Nutrition Care during Admission and After Discharge?

Poor nutrition, often worsened during hospitalization, is thought to contribute to a phenomenon termed “post-hospital syndrome”, which is a generalized transient vulnerability after hospital discharge which leads to higher morbidity and an increased rate of readmission for the same or other causes [72]. Continued nutritional support during hospitalization and at hospital discharge has been associated with reduced mortality, readmission, medical costs, and fewer discharges to a post-acute care facility in recent cohort studies of malnourished generalized populations [46,73,74]. In a recent large propensity-matched cohort study, Kaegi-Braun et al. found that the in-hospital mortality rate was significantly lower in patients who received nutritional support compared to those who did not (incidence rate ratio 0.79, 95% CI 0.75–0.84), with lower 30-day readmission rates [73]. Further studies focused specifically on IBD populations are warranted. 

In Alberta, Canada, a unique multidisciplinary High Risk Malnutrition Clinic led by gastroenterologists who are also physician nutrition specialists and dietitians was developed specifically to optimize nutrition care of malnourished patients with digestive diseases, including IBD [75]. This unique clinic incorporates comprehensive nutrition assessments and therapies as part of the regular treatment plan. Future studies will evaluate the efficacy of this approach on relevant clinical outcomes such as quality of life and hospital readmission. Gastroenterologists should play a crucial role in the identification, management, and arrangement of appropriate follow up of malnutrition including multidisciplinary care in the community upon discharge. 

## 8. Conclusions and Future Directions

Our narrative review highlighted high-priority areas for consideration in the nutritional management of hospitalized IBD patients (see Table 1 below). We outline knowledge gaps and limitations in the current literature related to malnutrition in IBD.

(A) *No gold standard definition of malnutrition in IBD and lack of validated NST and NAT*
  This applies to both hospitalized and community IBD populations. There is a wide range in the reported prevalence of malnutrition in IBD due to a lack of well-validated tools and wide variability in disease location and severity. A set of disease-specific yet encompassing criteria for malnutrition in IBD remains to be determined. Until further high-quality data are available, it is essential that all IBD patients admitted to hospitals be screened for malnutrition utilizing either the NRS-2002 or MUST NST with prompt nutritional interventions if at moderate to high risk of malnutrition.
(B) *Limited high-quality data for the use/timing of PN, including total versus supplemental, and central versus peripheral*
  While there are widely agreed-upon parameters for CPN use and timing, these are based on studies in non-IBD populations, using non-IBD specific nutritional risk assessment tools. Further studies of PN use, including timing and dosing, are warranted. There is a lack of data for when and how PPN can be optimized in patients with IBD. There are critical points early in hospital admission where patients may benefit from PPN as a bridge or supplement to longer-term therapy, but there are no data to guide this use. How IBD patients may benefit from PN requires further research.
(C) *Paucity of data on the impact of nutritional interventions on hospitalized IBD patients*
  While there is growing literature on the impact of nutrition interventions in improving IBD peri-operative risks, there remains a lack of high-quality studies for benefits of nutrition interventions on other outcomes of interest (infections, length of stay, readmission, surgery, etc.). Various nutritional therapies including ONS, EN, PPN, supplementary PN, and CPN were explored in this review, and recommendations for the sequenced use of nutritional therapies were provided based on data from other conditions.

In summary, we outlined the high prevalence of malnutrition in IBD arising from its unique pathophysiology and the importance of malnutrition screening in all hospitalized IBD patients. We provide an expert opinion on diagnosing malnutrition in IBD that does not require weight loss or low BMI. Finally, we suggest approaches to nutrition therapies for the hospitalized IBD patient. Future prospective studies should prioritize these important areas of research to advance our understanding of how to best treat malnutrition in IBD.

## Figures and Tables

**Table 1:** Take-home clinical points.

Key Points
Malnutrition is highly prevalent in IBD patients.
All hospitalized IBD patients should be screened for malnutrition. atrogenic factors contributing to malnutrition in hospitals should be minimized. Malnourished IBD patients should be treated with ONS, EN, PPN, CPN or some combination of these nutrition interventions.
